# Hypergravity Enhances Stretch Sensitivity in Rat Cardiomyocytes via Increased Expression and Activity of Stretch-Activated Channels

**DOI:** 10.3390/ijms26199284

**Published:** 2025-09-23

**Authors:** Andre G. Kamkin, Valentin I. Zolotarev, Olga Kamkina, Vadim M. Mitrokhin, Viktor E. Kazansky, Andrey Bilichenko, Anastasia S. Rodina, Alexandra D. Zolotareva, Mitko Mladenov

**Affiliations:** Institute of Physiology, Pirogov Russian National Research Medical University, Moscow 117997, Russia; andrey.kamkin@rsmu.ru (A.G.K.); zolotarev_vi@rsmu.ru (V.I.Z.); kamkina_ov@rsmu.ru (O.K.); mitrokhin_vm@rsmu.ru (V.M.M.); kazanskii_ve@rsmu.ru (V.E.K.); bilichenko_as@rsmu.ru (A.B.); rodina_as@rsmu.ru (A.S.R.); zolotareva_ad@rsmu.ru (A.D.Z.)

**Keywords:** hypergravity, cardiomyocytes, mechanically gated channels, mechanosensitive channels, Gene Transcripts, patch-clamp

## Abstract

Although hypergravity may influence cardiac mechanosensitivity, the effects on specific ion channels remain inadequately understood. This research examined the effects of long-term hypergravity on the functional activity and transcriptional expression of mechanosensitive channels (MSCs) in rat ventricular cardiomyocytes. After 14 days of exposure to 4g, rats were subjected to molecular and electrophysiological analyses. Significant remodeling of MSC-encoding genes was revealed by RNA-seq. *Trpm7* (+41.23%, *p* = 0.0073) and *Trpc1* (+68.23%, *p* = 0.0026) were significantly upregulated among non-selective cation channels, while *Trpv2* (−62.19%, *p* = 0.0044) and *Piezo2* (−57.58%, *p* = 0.0079) were significantly downregulated. *Kcnmb1* (−47.84%, *p* = 0.0203) was suppressed, whereas *Traak*/K2P4.1 showed a strong increase (+239.48%, *p* = 0.0092), among K^+^-selective MSCs. Furthermore, *Kir6.1* was significantly downregulated (−75.8%, *p* = 0.0085), whereas *Kir6.2* was significantly upregulated (+38.58%, *p* = 0.0317). These results suggest targeted transcriptional reprogramming that suppresses pathways associated with maladaptive Ca^2+^ influx while enhancing Ca^2+^-permeable mechanosensitive channels alongside stabilized K^+^ conductance. At the structural level, cardiomyocytes from hypergravity exposure showed a 44% increase in membrane capacitance, consistent with hypertrophic remodeling, and sarcomere elongation (*p* < 0.001). Functionally, stretch-activated current (*I*_SAC_) was markedly hypersensitive in patch-clamp analysis: currents were induced at very small displacements (1–2 µm) and were significantly larger under 4–10 µm stretch (222–107% of control values). These findings indicate that chronic hypergravity induces coordinated molecular, structural, and functional remodeling of cardiomyocytes, characterized by increased membrane excitability, compensatory stabilizing mechanisms, and enhanced Ca^2+^ signaling. This demonstrates the flexibility of cardiac mechanotransduction under prolonged gravitational stress, with potential implications for understanding cardiovascular risks, arrhythmias, and hypertrophy associated with altered gravity environments.

## 1. Introduction

The mechanical stimulus may trigger an electrophysiological response in cardiomyocytes, a characteristic usually termed mechanoelectric feedback, which was first reported by Kaufmann and Theophile (1967) and Lab (1968) [1,2,3]. This mechanism is predominantly achieved by transmembrane cation influxes via stretch-activated channels (SACs), which can alter the membrane potential and induce arrhythmogenesis under both physiological and pathological conditions [4,5,6].

Localized mechanical stimulation of single ventricular [7,8] and atrial [9,10] myocytes has been experimentally demonstrated to excite non-selective SACs and thereby generate inward currents that amplify cellular excitability. Ca_V_1.2 has been shown to be stretch-sensitive at the single-channel [11] and whole-cell levels in cardiomyocytes [12]. These *L*-type Ca^2+^ channels, and SACs, which transduce non-selective cation influx during membrane deformation, belong to the class of mechanosensitive channels (MSCs). A subset of these channels—the stretch-or-compression gated ones—are more properly called mechanically gated channels (MGCs) [8,11].

The family of MSCs includes not only SACs and *L*-type Ca^2+^ channels (e.g., Ca_V_1.2 and Ca_V_1.3) [12,13] but also inwardly rectifying potassium (K_ir_) channels and voltage-insensitive Na^+^ and K^+^ channels [14], which also possess sensitivity to mechanical stimuli. These channels are the major transducers of mechanical stimuli in the electrical response and the primary contributors to electromechanical coupling.

The responsiveness of cardiomyocytes to mechanical stress is not static but can be profoundly influenced by aging and disease-induced remodeling. Especially intriguing is the stretch-activated current (*I*_SAC_) that represents non-selective cation influx induced by mechanical distension of the sarcolemma. One comparative study demonstrated that Wistar-Kyoto (WKY) and spontaneously hypertensive rats (SHRs) showed an increase in *I*_SAC_ amplitude that corresponded with cellular hypertrophy and elevated membrane capacitance [7]. Under a Cs^+^_in_/Cs^+^_out_ configuration in control conditions (to eliminate other K^+^ currents), *I*_SAC_ was significantly augmented in aged and hypertrophic rats [7,8,14,15]. It was especially intriguing that an 8 µm stretch induced a small inward current in young WKY myocytes but led to strong responses in aged WKY and SHR cells, suggesting mechanical hypersensitivity as a result of pathological structural remodeling [7,16]. Such observations indicate that disease states such as hypertension and aging can modify mechanotransduction pathways, probably through changes in channel expression, membrane–cytoskeletal coupling, or submembranous architecture. In addition, recent evidence suggests that changes in gravity (microgravity and hypergravity) may also affect cardiac physiology [17], altering heart mass and morphology [18], calcium handling [17], and mechanotransduction-dependent transcription of mechanosensitive genes [19].

Given that precisely controlled Ca^2+^, Na^+^, and K^+^ fluxes are crucial for cardiomyocyte function [20], together with several transporters across the cell membrane [21], it is essential to understand how these components are affected by gravity. However, cardiac adaptation to hypergravity, with modifications of MSCs and concomitant electrophysiological remodeling, is still poorly understood.

We distinguish between the principal subtypes of mechanosensitive ionic conductance. Especially interesting is the stretch-activated current (*I*_SAC_) that represents non-selective cation influx induced by mechanical distension of the sarcolemma. We also investigate the late Ca^2+^ current (*I*_L_)—a sustained inward current that follows the transient *L*-type Ca^2+^ current peak. *I*_L_ acts as a sensitive substrate for sustained Ca^2+^ entry or slow Ca^2+^ buffering, which are frequently affected by mechanical stimuli or redox alteration. A common measure of Ca^2+^ handling is the amplitude, which can be used to gauge the subtle effects of mechanical stress and pharmacologic interventions.

Based on the above, the present study was designed to examine the effects of 14 days of hypergravity exposure on mechanoelectric feedback (MEF) in rat ventricular cardiomyocytes. Specifically, we investigated whether hypergravity:

(i) enhances *I*_SAC_ sensitivity to mechanical distension;
(ii) regulates the transcriptional expression of MGC- and MSC-related genes; and
(iii) enhances stretch-induced cation conductance.

We hypothesized that hypergravity may stimulate structural and transcriptional adaptations, leading to mechanoelectrical “tuning” of the myocyte. This knowledge may provide insight into how cardiac adaptation is initiated in an altered gravitational environment.

## 2. Results

### 2.1. Alterations in RNA Transcript Levels Under Hypergravity Conditions

**Figure 1:**
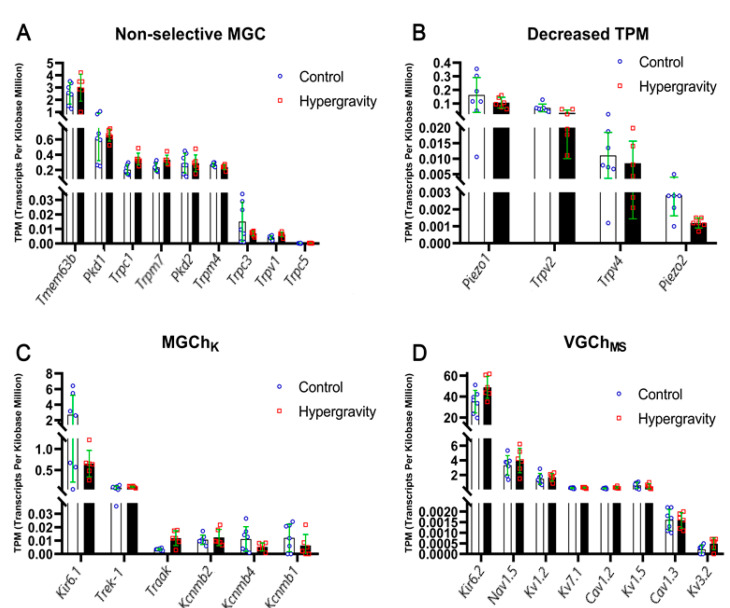
Changes in RNA transcript levels, calculated in TPM, for non-selective mechanically gated channels (**A**), channels with decreased TPM (**B**), K^+^-selective mechanically gated channels (**C**), and mechanosensitive ion channels (**D**) in ventricular cardiomyocytes of rats under control conditions (white bars) and after 14 days of hypergravity exposure (black bars).

Gene expression profiling following 14 days of hypergravity revealed restricted but significant remodeling of MSCs in ventricular cardiomyocytes. The results indicate a defined set of channels that underwent robust transcriptional regulation (Table 1, Figure 1).

#### 2.1.1. Non-Selective Cation MGCs

Among the TRP and related MSCs, *Trpm7* and *Trpc1* exhibited significant upregulation. *Trpm7* increased by 41.2% (*p* = 0.0073), consistent with its established role in Ca^2+^/Mg^2+^ homeostasis and mechanotransduction. Similarly, *Trpc1* increased by 68.2% (*p* = 0.0026), supporting its role in mechanically induced Ca^2+^ influx and calcineurin–NFAT-dependent transcription. In contrast, *Trpv2* was reduced by 62.2% (*p* = 0.0044), suggesting its involvement in cardiomyocyte adaptation and prevention of Ca^2+^ overload. In addition, *Piezo2* expression was significantly decreased by 57.6% (*p* = 0.0079), indicating a selective conductance refinement of cardiac mechanosensitive pathways.

#### 2.1.2. K^+^-Selective MGCs

Among the two-pore domain K^+^ channels, *Traak* (K2P4.1) displayed a highly significant change, increasing by 239.5% (*p* = 0.0092). As a channel that drives hyperpolarization, TRAAK channels induce membrane potential stabilization under conditions of stretch. Indeed, its upregulation indicates an electrophysiological counterbalance to the enhanced cation influx mediated by TRPM7 and TRPC1. Among the BK_Ca_ channel subunits, *Kcnmb1* was significantly reduced (−47.8%, *p* = 0.0203), reflecting the altered regulation under hypergravity conditions.

#### 2.1.3. Mechanosensitive Voltage-Gated and Other Channels

Among voltage-gated and K_ATP_-related channels, two additional significant modifications were detected. The inward rectifier *Kir6.2* was significantly upregulated (+38.6%, *p* = 0.0317), while its closely related isoform *Kir6.1* was strongly downregulated (−75.8%, *p* = 0.0085). This reciprocal remodeling of K_ATP_ channel subunits suggests a shift in channel composition that could modify ATP sensitivity and mechano-metabolic coupling.

Figure 1 illustrates these significant transcript changes, with pronounced upregulation of *Trpm7*, *Trpc1*, *Traak*, and *Kir6.2*, and strong suppression of *Trpv2*, *Piezo2*, *Kir6.1*, and *Kcnmb1*. Collectively, these alterations indicate that hypergravity induces a targeted transcriptional program, enhancing Ca^2+^-permeable mechanosensitive channels and stabilizing K^+^ conductance, while simultaneously downregulating channels linked to maladaptive Ca^2+^ entry.

### 2.2. The Impact of Hypergravity Exposure on Sarcomere Length (SL) in the Cardiomyocytes

The ventricular myocytes from young rats had an average length of 125 ± 8 µm and a width of 25 ± 5 µm. Optical imaging was the primary tool for characterizing the sarcomeric striation pattern and the corresponding average sarcomere length (SL), which is crucial for understanding the functional relationships between actual SL and the activity of MGCs under hypergravity conditions.

The SL of each isolated cardiomyocyte was calculated using the distance between sarcomeres along a central line in the cell. Curved myofibrils, crossing myofibrils, or myofibrils with irregular staining were omitted from the analysis. SL was estimated in isolated cardiomyocytes by measuring the distance between ten sarcomeres along the line connecting sarcomeric S and P bands.

The average SL in control animals, measured using light microscopy and fluorescence microscopy with membrane staining ANEPPS, was 1.83 ± 0.005 µm (n = 16; number of measured lines between S and P bands for ten sarcomeres: L = 16; m = 4). In ventricular cardiomyocytes obtained from rats exposed to hypergravity, the SL was 1.96 ± 0.01 µm (*p* < 0.001 compared to control; n = 10; L = 20; m = 4).

### 2.3. The Impact of Hypergravity Exposure on Trpm7 Protein Expression in Cardiomyocytes

To determine whether hypergravity-induced transcriptional changes translate to the protein level, we assessed *Trpm7*, a representative MGC, in rat ventricular cardiomyocytes following 14 days of hypergravity exposure.

**Figure 2:**
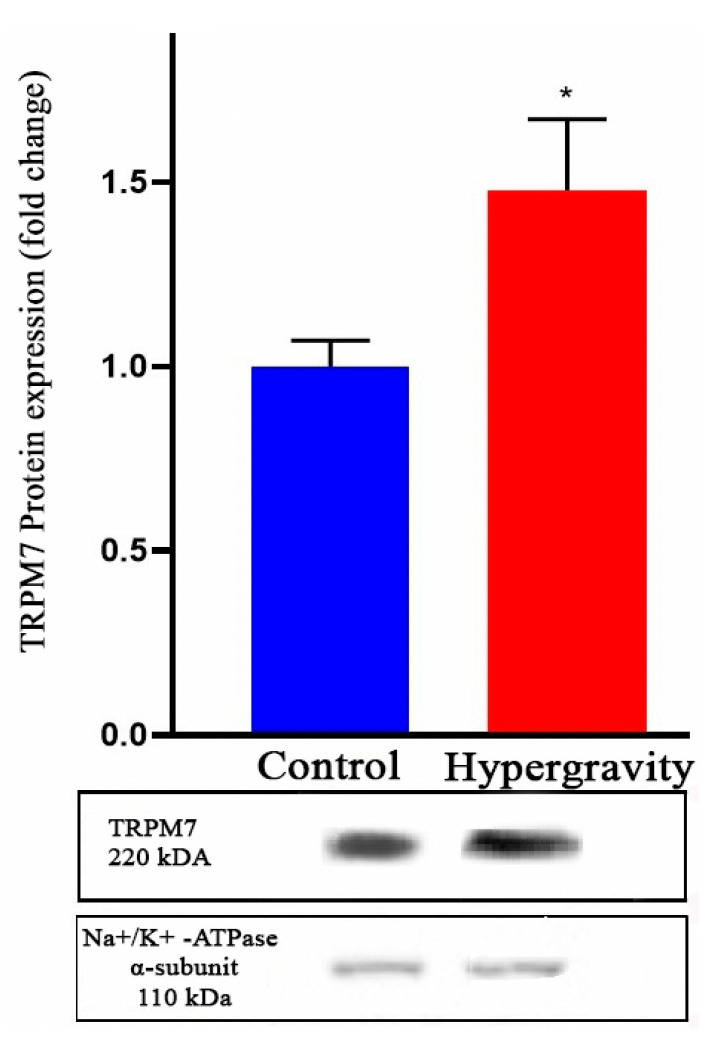
Hypergravity increases TRPM7 protein expression in rat ventricular cardiomyocytes. Representative immunoblots and quantitative analysis of TRPM7 protein levels in isolated ventricular myocytes from control rats (n = 11) and rats exposed to hypergravity (4 g for 14 days, n = 10). Protein expression was normalized to the Na^+^/K^+^-ATPase α-subunit. Data are expressed as mean ± SEM. * *p* < 0.01 vs. control.

Western blot analysis revealed a significant increase in *Trpm7* protein expression in the hypergravity group compared to controls (Figure 2). When normalized to the Na^+^/K^+^-ATPase α-subunit (used as a housekeeping control), *Trpm7* levels increased from 1.00 ± 0.25 in control myocytes (n = 11) to 1.48 ± 0.19 in hypergravity-exposed cells (n = 10; *p* < 0.01).

These findings indicate that hypergravity enhances both *Trpm7* mRNA (TPM) and protein expression, suggesting a coordinated transcriptional and translational response. Notably, this molecular upregulation is consistent with our electrophysiological data, which demonstrate increased *I*_SAC_ activity in cardiomyocytes from hypergravity-exposed rats. Collectively, these results support the hypothesis that hypergravity modulates the expression of genes for MSCs, resulting in elevated protein abundance and enhanced mechanically gated ionic conductance, thereby increasing cellular mechanosensitivity.

### 2.4. The Impact of Stretch upon Sarcomere Length (SL) in the Cardiomyocytes

Local cell stretching was performed along the line connecting the glass stylus (S) and patch pipette (P), which were approximately 40 ± 1 µm apart, constituting about 32% of the cell length. As previously shown, under control conditions, before stretching, the sarcomere length (SL) in isolated rat ventricular myocytes was 1.83 ± 0.005 µm, estimated from the distance between ten sarcomeres along the S-P line. In ventricular myocytes from intact rats, neither the touch of the S (n = 5, L = 10) nor local cell stretching by 1 µm (n = 5, L = 10) or 2 µm (n = 5, L = 10) led to any changes in SL. Additionally, these actions did not induce *I*_SAC_ at −80 mV or cause changes in the current/voltage (*I*/*V*) curves of the *I_L_*.

However, stretching the cell by 4 µm increased SL by approximately 6% to 1.92 ± 0.01 µm (n = 5, L = 10, m = 3), by 6 µm by about 10% to 2.02 ± 0.01 µm (n = 5, L = 10, m = 3), by 8 µm by about 14% to 2.10 ± 0.01 µm (n = 5, L = 10, m = 3), and by 10 µm by about 18% to 2.15 ± 0.01 µm (n = 5, L = 10, m = 3).

In ventricular cardiomyocytes obtained from rats exposed to hypergravity, the SL was initially 1.96 ± 0.01 µm. Cell stretching by 4 µm increased SL by approximately 6% to 2.07 ± 0.01 µm (n = 5, L = 10, m = 3), by 6 µm by about 10% to 2.15 ± 0.01 µm (n = 5, L = 10, m = 3), by 8 µm by about 14% to 2.23 ± 0.01 µm (n = 5, L = 10, m = 3), and by 10 µm by about 18% to 2.31 ± 0.01 µm (n = 5, L = 10, m = 3).

The data demonstrate that although the initial SL in control and hypergravity-exposed cardiomyocytes differed, stretching the cells by 4, 6, 8, and 10 µm proportionally increased SL in both groups of cells by 6%, 10%, 14%, and 18%, respectively.

### 2.5. Stretch Sensitivity of I_SAC_ in the Cardiomyocytes

#### 2.5.1. I_SAC_ in the Cardiomyocytes from the Control Rats

Cells with similar geometry, selected based on their length and diameter, had an average membrane capacitance of 149 ± 2.0 pF (n = 103). These data are consistent with previously reported values [7,14].

In cardiomyocytes from control animals, mechanical manipulations, including moving the patch-pipette by 1 µm (n = 12), stylus adhesion (n = 12), or stretching the cell by 2 µm using the stylus (n = 14), did not lead to any changes in *I_L_*, indicating no current through MGCs (m = 10 hearts for these three series of experiments). The appearance of current through MGCs was only registered with local stretching of the area between S and P by 4 µm, although even at this level of stretching, currents through MGCs did not always occur.

**Figure 3:**
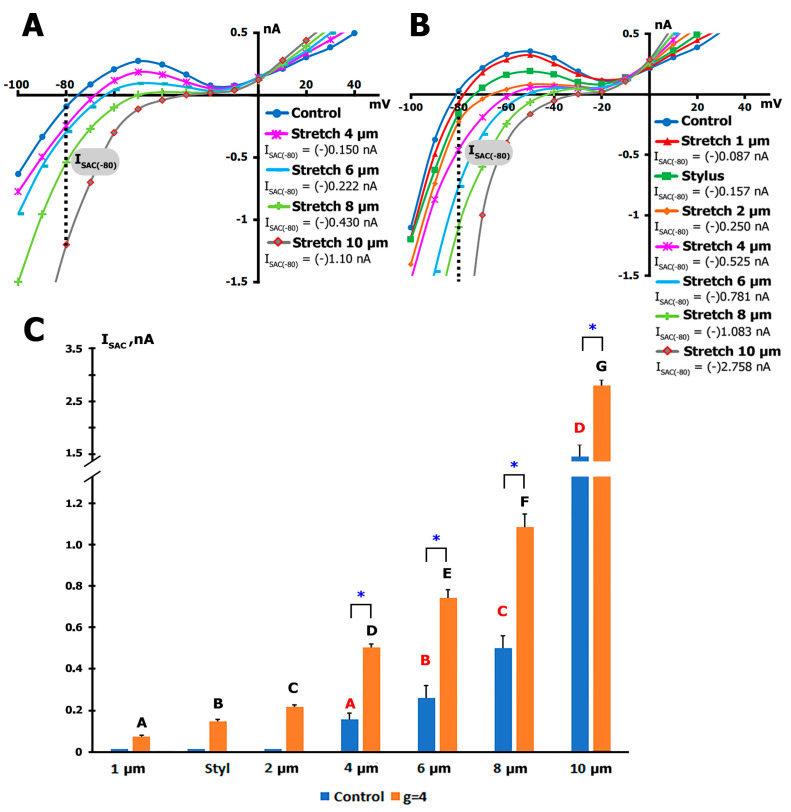
Hypergravity in rats increases the sensitivity of mechanically gated currents to stretch. (**A**) Increase in *I_L_* at −80 mV in K^+^_in_/K^+^_out_ solutions during local stretching of cardiomyocytes from control rats by 4, 6, 8, and 10 µm. *V_hp_* = −45 mV. *I*/*V* curve of *I_L_* before (blue circles) and during stretching by 4 µm (red triangles), 6 µm (green squares), 8 µm (orange diamonds), and 10 µm (purple stars). Cell capacitance was 170 pF. (**B**) Increase in *I_L_* at −80 mV in K^+^_in_/K^+^_out_ solutions in rats exposed to hypergravity during control (blue circles) and local stretching of cardiomyocytes by 1 µm (red triangles), stylus touch (green squares), 2 µm (orange diamonds), 4 µm (purple stars), 6 µm (light blue strokes), 8 µm (light green crosses), and 10 µm stretch (gray diamonds). *V_hp_* = −45 mV. Cell capacitance was 170 pF. (**C**) The effect of discrete stretching of cardiomyocytes on the magnitude of *I_SAC_* in cells from both control animals and those subjected to hypergravity. Note the break in the coordinate axis indicating the current amplitude in pA and the different scales after the axis break. (A–G) Different experimental groups compared using post hoc statistical analysis. Groups not sharing the same letter are significantly different from each other (*p* < 0.05). * Indicates statistically significant difference between control and *g* = 4 groups at the corresponding stretch (*p* < 0.05).

In cells from control animals, the *I*/*V* curves in Figure 3A show the voltage dependence of *I_L_* and its modulation by 4, 6, 8, and 10 µm stretch in K^+^_in_/K^+^_out_ solutions. Before stretching (n = 8, m = 3), the *I*_L_ *I*/*V* curve was *N*-shaped and crossed the voltage axis (zero current potential *V*_0_) at −76 mV (−76  ±  2 mV), equivalent to the resting potential of the non-clamped cell.

Figure 3A shows that the magnitude of *I*_L_ at −80 mV in control conditions is −0.111 nA. A modest stretch of 4 µm (n = 10, m = 4 hearts) shifted the net currents *I*_L_ at −80 mV to more negative values, from *^C^I_L_* = −0.111 nA to *^S^I_L_* = −0.260 nA. In this case, *I*_SAC_ was −0.156 nA (−0.156  ±  0.03 nA). The *V*_0_ shifted to a more depolarized area from −76 mV (−78  ±  2 mV) to −69 mV (−66  ±  3 mV) with the stretch.

Stretching by 6 µm (n = 8, m = 4 hearts) shifted the net currents *I_L_* at −80 mV even further to negative values to *^S^I_L_*= −0.331 nA. In this case, *I*_SAC_ was −0.222 nA (−0.26  ±  0.06 nA). The *V*_0_ also shifted to −63 mV (−60  ±  3 mV) with the stretch.

Stretching by 8 µm (n = 6, m = 5 hearts) shifted *I_L_* at −80 mV to *^S^I_L_* = −0.541 nA. In this case, *I_SAC_* was −0.43 nA (−0.50  ±  0.06 nA). The *V_0_* also shifted to −50 mV (−52.7  ±  3 mV) with the stretch.

Stretching by 10 µm (n = 5, m = 8 hearts) shifted *I_L_* at −80 mV to *^S^I_L_* = −1.211 nA. In this case, *I*_SAC_ was −1.10 nA (−1.33  ±  0.11 nA). The *V_0_* also shifted to −30 mV (−34  ±  3 mV) with the stretch.

#### 2.5.2. I_SAC_ in the Cardiomyocytes from the Hypergravity-Exposed Rats

In ventricular cardiomyocytes from rats exposed to hypergravity, the magnitude of *I_SAC_* during cell stretching significantly increased (Figure 3B). Measurements of *^C^I_L_* and *^S^I_L_* (with subsequent calculation of *I_SAC_*) under mechanical stress, associated with adhesion of S to the cell and discrete cell stretching of 1, 2, 4, 6, 8, and 10 µm, demonstrated enhanced sensitivity of the cardiomyocytes to stretch (Figure 3B).

In ventricular cardiomyocytes from healthy young control rats, *I_SAC_* never appeared with a 1 µm displacement of the patch-pipette. However, in cells from experimental rats, this action led to the appearance of *I*_SAC_ with an amplitude of −0.087 nA (−0.073 ± 0.006 nA) and a shift of *V*_0_ to a more depolarized region from −82 mV (−80 ± 3 mV) to −78 mV (−77 ± 3 mV). These experiments involved (n = 8, m = 5) hearts.

Even more surprising was the occurrence of *I*_SAC_ upon the adhesion of the stylus to the cell surface. While ensuring satisfactory contact during stretching, the stylus slightly pressed the cell by approximately 1 µm (Figure 3B), but this never caused the appearance of mechanically induced currents or any changes in the *I*/*V* curve in control cells. However, in cells from animals subjected to hypergravity, interaction with the cell surface by the stylus (n = 10, m = 6) caused *I*_SAC_ with a magnitude of −0.157 nA (−0.147 ± 0.011 nA). Simultaneously, *V*_0_ shifted to a more depolarized region to −73 mV (−72 ± 2 mV).

Subsequent stretching of the cell using the stylus by 2 µm (n = 16, m = 6) caused *I*_SAC_ with a magnitude of −0.250 nA (−0.218 ± 0.010 nA), relative to control values. This resulted in a shift of *V*_0_ to the depolarized region at −65 mV (−66 ± 2 mV). Further stretching of the cell by 4 µm (n = 6, m = 4) caused *I*_SAC_ with a magnitude of −0.525 nA (−0.503 ± 0.015 nA), relative to control values. This resulted in a shift of *V*_0_ to the depolarized area at −58 mV (−56 ± 3 mV). Stretching the cell by 6 µm (n = 5, m = 4) caused *I*_SAC_ with a magnitude of −0.781 nA (−0.742 ± 0.038 nA). This resulted in a shift of *V*_0_ to the depolarized region at −44 mV (−47 ± 3 mV). Stretching the cell by 8 µm (n = 5, m = 5) caused *I*_SAC_ with a magnitude of −1.083 nA (−1.124 ± 0.112 nA). This procedure resulted in a shift of *V*_0_ to the depolarized region at −44 mV (−47 ± 3 mV). In two instances, we recorded *I*_SAC_ during cell stretching by 10 µm (n = 2, m = 6), with a magnitude of −2.758 ± 0.180 nA.

The membrane capacitance of cardiomyocytes from hypergravity-exposed rats was significantly greater than in controls, averaging 183.9 ± 25.2 pF (n = 52) versus 127.4 ± 15.8 pF (n = 103) in the control group (*p* < 0.0001), indicating cell enlargement consistent with hypertrophic remodeling.

#### 2.5.3. Comparison of I_SAC_ Sensitivity to Stretch in Cardiomyocytes from Control and Hypergravity-Exposed Rats

Figure 3C demonstrates the occurrence of *I_SAC_* during the stretching of a cardiomyocyte from rats exposed to hypergravity by a 1 µm displacement of the patch-pipette and stretching by 2 µm using the stylus—actions that never induce changes in control animals. In ventricular myocytes from hypergravity-exposed rats, stretching by 4 µm resulted in an *I_SAC_* that was 222% greater than in control animals. Stretching by 6 µm caused an *I_SAC_* that was 185% greater than in control animals. At 8 µm of stretch, *I_SAC_* increased by 124% compared to control cells, and at 10 µm of stretch, *I_SAC_* increased by 107%.

Stretching cardiomyocytes from experimental animals by 2 µm induced a current comparable to that generated by stretching cardiomyocytes from control animals by 6 µm. Similarly, stretching cardiomyocytes from experimental animals by 4 µm induced a current comparable to that generated by stretching cardiomyocytes from control animals by 8 µm.

## 3. Discussion

The present work demonstrates that prolonged hypergravity induces a complex remodeling process in cardiomyocytes, encompassing structural, functional, and transcriptional alterations. MSCs undergo selective molecular reprogramming, leading to the inhibition of maladaptive conductances and the enhanced expression of Ca^2+^-permeable and stabilizing K^+^ channels. The following sections present in detail how these transcriptional modifications align with structural alterations and mechanoelectric feedback.

### 3.1. Mechanosensitive Ion Channel Remodeling and Structural Adaptations Under Hypergravity

#### Coordinated Ion Channel Remodeling in Mechanically Loaded Cardiomyocytes

Our research demonstrates that prolonged exposure to hypergravity induces a selective yet coordinated remodeling of MSCs in ventricular cardiomyocytes. Several channels demonstrated statistically significant alterations, indicating a specific molecular response rather than a broad stress response.

The most significant changes were observed in *Trpm7* (+41.2%, *p* = 0.0073) and *Trpc1* (+68.2%, *p* = 0.0026), both of which are crucial regulators of Ca^2+^ influx in response to mechanical stress. *Trpm7* regulates Mg^2+^ and Ca^2+^ concentrations and modulates signals for hypertrophic development [72]. *Trpc1* regulates the influx of Ca^2+^ induced by mechanical stretching, which stimulates the calcineurin—nuclear factor of activated T-cells (NFAT) pathway, thereby facilitating transcriptional response to mechanical strain [73,74]. The simultaneous increase of these channels clearly suggests that hypergravity amplifies Ca^2+^-dependent mechanotransduction, facilitating long-term structural and functional remodeling [73,74,75].

The significant downregulation of *Kcnmb1* (−47.8%, *p* = 0.0203) found in our study occurred in conjunction with extremely low baseline expression of the pore-forming subunit *Kcnma1* (0.0001 ± 0.0001 TPM). As such, both control and hypergravity conditions were comparable in this respect. Although it may seem surprising at first glance, this pattern reflects the particular manner in which BK_Ca_ channels work in adult heart tissue and throws light on various mechanisms of adaptation to hypergravity.

The fact that *Kcnma1* is almost totally absent from our isolated ventricular myocytes is consistent with the literature, which has indicated that it is a characteristic of heart cell development. Actually, BK_Ca_ channels in the heart are mostly located on the mitochondrial membrane, and they perform specialized protective functions for the heart muscle. This tissue-specific expression pattern creates a physiological scenario where traditional heteromeric BK_Ca_ channel complexes (α_4_β_4_) are largely absent from the cardiomyocyte sarcolemma.

The relatively higher *Kcnmb1* expression, along with the minimal *Kcnma1* levels, reflects several important biological functions independent of classical BK_Ca_ channel formation within cardiomyocytes. In this direction, the work of Yang et al. (2009) indicates that KCNMB subunits possess independent functions [76]. Actually, they have shown that KCNMB subunits can specifically tune other potassium channels, such as the Slo3 channel, where only the β-subunit can boost conductance up to 8-fold while showing no interaction with KCNMA subunits [76]. In addition, it was shown that β-subunits serve a scaffold or regulatory function for other membrane protein complexes through their membrane-spanning topology. Analysis of BK_Ca_-associated proteins revealed 110 putative protein partners in cochlear tissue, suggesting extensive protein interaction networks that could function independently of channel formation [77].

Under hypergravity conditions, the reduction in *Kcnmb1* is therefore a measurable molecular response that reflects the downregulation of non-canonical β-subunits’ function in myocytes. This finding indicates that hypergravity causes a targeted remodeling of the auxiliary channel regulatory mechanisms, which reveals the sophisticated molecular adaptations that occur during sustained gravitational stress.

There was a significant increase in *Traak*/K2P4.1 (+239.5%, *p* = 0.0092), a potassium channel responsive to lipids and mechanical stimuli [78,79]. *Traak* activation produces outward hyperpolarizing currents that counteract depolarizing cation influx through TRPs, serving as an intrinsic “safety valve” against Ca^2+^-mediated cytotoxicity. This robust upregulation indicates that hypergravity enhances electrophysiological stability during sustained mechanical stress, corroborating earlier research demonstrating that K2P channels are essential for mechanoelectric feedback [59,79,80].

Conversely, *Trpv2* (−62.2%, *p* = 0.0044) and *Piezo2* (−57.6%, *p* = 0.0079) exhibited substantial downregulation. Excessive activation of *Trpv2* has been linked to pathological calcium overload and dilated cardiomyopathy [30]. Thus, its downregulation under hypergravity may represent a cytoprotective response. *Piezo2* is crucial for sensory mechanotransduction and baroreceptor signaling [55,56]; however, its functions in cardiomyocytes are largely unknown. This reduction likely signifies a “filtering” mechanism that refines the cardiac mechanosensitive repertoire towards channels more directly implicated in cardiac adaptation.

Hypergravity also altered the balance of ATP-sensitive K^+^ channel subunits. The expression of *Kir6.2* increased (+38.6%, *p* = 0.0317), whereas the expression of *Kir6.1* decreased (−75.8%, *p* = 0.0085). K_ATP_ channels are tetrameric structures composed of Kir6.x subunits and Sulfonylurea Receptor (SUR) partners [70,71]. These alterations in opposite directions may modify the subunit composition, leading to a prevalence of Kir6.2-dominant assemblies [70]. Such alterations may reduce ATP sensitivity, facilitating channel opening under metabolic stress and aiding in adaptation to hypergravity by regulating cardiac electrical activity.

These modifications indicate that hypergravity triggers a dual remodeling process:

1. Enhances calcium signaling pathways through *Trpm7* and *Trpc1*, thereby preserving mechanoelectrical integration and transcriptional flexibility.
2. Promoted protective electrical stability (via *Traak* and *Kir6.2* overexpression and *Trpv2*/*Piezo2* downregulation) to prevent arrhythmogenic risk.

This precisely coordinated remodeling is consistent with the fundamental principle of the heart’s response to mechanical stress and illustrates the adaptability of cardiac mechanosensing under sustained gravitational pressure.

### 3.2. Structural Remodeling and Sarcomere Adaptation Under Hypergravity

We examined sarcomere lengths (SL) and membrane capacitance in freshly isolated ventricular myocytes to assess whether hypergravity-induced transcriptional changes were associated with structural remodeling. Resting SL was 1.83 ± 0.01 µm in control animals (a value consistent with previous literature in adult rats [81]). In hypergravity-exposed myocytes, SL was significantly longer (1.96 ± 0.01 µm, *p* < 0.001), indicating that the sarcomeres had elongated under the influence of hypergravity exposure.

To differentiate passive stretch from hypertrophic remodeling, we assessed membrane capacitance as an indirect measure of cell size. Myocytes from hypergravity-exposed animals demonstrated a 44% increase in capacitance compared to control cells (183.9 ± 25.2 pF vs. 127.4 ± 15.8 pF; *p* < 0.0001), indicative of cellular enlargement and suggesting structural hypertrophy.

Age-related hypertrophy in rats is associated with increases in cell size, with no significant change in SL [81,82,83]. The increase in sarcomere length observed here might instead represent an independent adaptive process consistent with the Frank–Starling mechanism, in which increased ventricular volume stretches the sarcomeres, enhancing their contractile effectiveness [84,85]. Chronic hypergravity exposure might function similarly to physical training, causing the sarcomeres to elongate over time for stronger force production under constant stress.

### 3.3. Enhanced Sensitivity of I_SAC_ and Mechanotransductive Amplification

Functional patch-clamp analysis demonstrated that the transcriptional remodeling induced by hypergravity is associated with a significant enhancement of *I*_SAC_. In control cardiomyocytes, displacements of 1–2 µm did not elicit measurable *I*_SAC_; however, in hypergravity-conditioned myocytes, the same minimal stimuli induced significant inward currents (−0.087 nA and −0.250 nA, respectively). At greater displacements, *I*_SAC_ amplitudes increased by 222% (4 µm), 185% (6 µm), and 124% (8 µm) compared to controls. These data show that hypergravity induces a significant decrease in the activation threshold of MSCs.

The molecular findings provide a mechanistic explanation. The enhanced current magnitude at lower stretches is likely the result of increased expression of *Trpm7* and *Trpc1*, which expands the pool of Ca^2+^-permeable channels. In parallel, upregulated *Traak* provides counterbalancing outward K^+^ flux, which may stabilize excitability despite greater cation entry. The close correlation between *Traak* expression and *I*_SAC_ amplitude suggests that *Traak* is an important modulator of stretch sensitivity. Conversely, the downregulation of *Trpv2* decreases the probability of pathological Ca^2+^ overload, indicating a hypergravity-driven adaptive recalibration of mechanosensitive signaling.

Structural remodeling, in addition to transcriptional alterations, also played a significant role in this hypersensitivity. Cardiomyocytes from rats exposed to hypergravity showed increased sarcomere length and a ~44% increase in membrane capacitance, consistent with hypertrophic growth. This type of cytoskeletal and sarcolemmal remodeling enhances the mechanical coupling between the lipid bilayer and channel proteins, which promotes channel activation at lower levels of stretch [79,85,86,87]. This phenomenon mirrors observations in hypertrophic and senescent myocardium, wherein cytoskeletal stiffening enhances SAC activity [86].

Indeed, the observed mechanotransductive enhancement may play a dual role. First, it maintains cardiomyocytes’ mechanical sensitivity even in the mechanically overloaded state, improving adaptive contractility and mechanoelectric feedback [86]. Second, enhanced SAC activity carries a potential risk for arrhythmogenesis if counterbalancing mechanisms such as Traak are overwhelmed [79].

Thus, hypergravity stimulates a remodeling program that decreases the mechanical threshold for *I*_SAC_ activation while simultaneously stabilizing electrical activity. This dual adaptation increases the heart’s capacity for fine-tuned plasticity under sustained gravitational stress, but it also suggests potential vulnerabilities—particularly under conditions of additional stress or pathological remodeling. These findings suggest that further investigations are needed to be able to clarify channel-specific contributions.

## 4. Materials and Methods

### 4.1. Animals

The experiments were conducted in accordance with the Guide for the Care and Use of Laboratory Animals (8th edition, 2011) published by the US National Institutes of Health. The experimental protocol was approved by the Ethics Committee of the Russian National Research Medical University. Male outbred Wistar rats, aged 8 weeks and weighing between 180 and 200 g, were used for the experiments. The rats were housed under a 12:12 h light–dark cycle and had ad libitum access to food.

### 4.2. Generation of Hypergravity Conditions

Hypergravity conditions were artificially created using a centrifuge designed as an overload simulator, equipped with two arms, each 60 cm long, intended for use with rodents [88]. At the ends of these arms, freely hanging cages containing the rats deviated during the rotation of the centrifuge shaft. A constant counterclockwise rotation generated hypergravity of 4 g. The rats were exposed to hypergravity for 14 days, 8 h per day (from 09:00 to 17:00 h). The control group of animals was housed in the same room, with all animals having continuous access to food and water. The room temperature was maintained at 24 °C with a 12 h light–dark cycle. After 14 days under hypergravity and control conditions, the weight of the animals in both groups increased, ranging from 215 to 235 g.

### 4.3. Solutions

Ca^2+^-free physiological salt solution (Ca^2+^-free PSS) contained the following components (in mmol/L): 118 NaCl, 4 KCl, 1 MgCl_2_, 1.6 NaH_2_PO_4_, 24 NaHCO_3_, 5 sodium pyruvate, 20 taurine, and 10 glucose, adjusted to pH 7.4 with NaOH and bubbled with carbogen (95% O_2_ + 5% CO_2_) [8]. The enzyme medium was prepared by supplementing Ca^2+^-free PSS with 10 μmol/L CaCl_2_, 0.2 mg/mL collagenase (Type II, Worthington, 225 units/mg), and 1 mg/mL bovine serum albumin (Sigma-Aldrich, St. Louis, MO, USA) [8].

Before the actual experiments, cells were stored for at least 2 h in a modified Kraftbrühe (KB) medium containing (in mmol/L): 50 L-glutamic acid, 30 KCl, 3 MgSO_4_·7H_2_O, 20 taurine, 10 glucose, 30 KH_2_PO_4_, 0.5 EGTA, and 20 HEPES, adjusted to pH 7.3 with KOH [8,89]. The isolated cells were stored in KB medium for up to 8 h. Ventricular cardiomyocytes were perfused with a solution containing (in mmol/L): 150 NaCl, 5.4 KCl, 1.8 CaCl_2_, 1.2 MgCl_2_, 20 glucose, and 5 HEPES, adjusted to pH 7.4 with NaOH (K^+^_out_ solution). The internal pipette solution contained (in mmol/L): 140 KCl, 5 Na_2_ATP, 5 MgCl_2_, 0.01 EGTA, and 10 HEPES/KOH at pH 7.3 (K^+^_in_ solution). Throughout the text, this configuration is referred to as K^+^_in_/K^+^_out_ solutions. The pipette and bath solutions K^+^_in_/K^+^_out_ likely preserve the Na^+^ and Ca^2+^ gradients required for reverse or forward Na^+^/Ca^2+^ exchanger (NCX) activity, depending on membrane potential. Local ionic Ca^2+^ buffering (e.g., EGTA in the pipette) was employed to prevent cytosolic Ca^2+^ buildup regardless of NCX activity.

### 4.4. Isolated Cardiomyocyte Preparation

We followed a modified cell isolation procedure as previously described by Kamkin et al. [7,8]. Rats were anesthetized with an intraperitoneal injection of 80 mg/kg ketamine and 10 mg/kg xylazine, along with heparin (1000 U/kg) to prevent blood coagulation in the coronary vessels of the excised heart. The chest was opened, and the heart was quickly excised and attached to a Langendorff apparatus. A constant flow of 1 mL/min at 37 °C was used to flush the coronary vessels with carbogen-bubbled, Ca^2+^-free PSS for 5 min.

Following the initial perfusion, the hearts were retrogradely perfused with the same PSS, now supplemented with Worthington type II collagenase (0.5 mg/mL), 1 mg/mL bovine serum albumin (Sigma), and 10 µmol/L CaCl_2_ for 18–20 min. The perfusate was continuously bubbled with carbogen (95% O_2_–5% CO_2_), and the temperature was maintained at 37 °C. After enzymatic digestion, the enzymes were washed out with a modified KB medium [16], and the heart was disconnected from the perfusion system.

The ventricles were then excised and cut into strips 3 mm wide, which were held by the tip with tweezers and vigorously shaken in the KB solution to release the cells into the KB medium. The resulting cell suspension was filtered and stored in the KB medium at 22 °C until further use.

### 4.5. RNA Isolation, Sequencing, and Analysis

RNA was isolated directly from the cardiomyocytes using TRIzol (Invitrogen, Thermo Fisher Scientific, Inc., Dreieich, Germany) and subsequently subjected to chloroform extraction (Sigma-Aldrich, Schnelldorf, Germany), following the manufacturer’s instructions. The concentration and purity of RNA were assessed using a NanoDrop spectrophotometer (Thermo Fisher Scientific, Inc., Dreieich, Germany).

The isolated RNA was further purified using the RNeasy Mini Kit (Qiagen, Hilden, Germany), adhering to the manufacturer’s protocol. RNA quantity and quality were re-evaluated using the NanoDrop spectrophotometer and the Qi-RNA kit (Thermo Fisher Scientific, Inc., Dreieich, Germany). Subsequently, samples were prepared with the NEB Ultra II RNA kit (New England Biolabs, Ipswich, MA, USA) following the provided instructions, incorporating the NEBNext Poly(A) Magnetic Isolation Module for mRNA (New England Biolabs, Ipswich, MA, USA) and unique dual-indexing.

The concentration, size distribution, and quality of the resulting libraries were evaluated using a Qubit 4 fluorometer (Thermo Fisher Scientific, Inc., Dreieich, Germany) with a High-Sensitivity dsDNA kit (Invitrogen, Carlsbad, CA, USA), and a 4200 TapeStation with a High-Sensitivity D5000 kit (Agilent, Santa Clara, CA, USA). Based on these assessments, libraries were normalized according to their molarity, pooled, and then quantified using a library quantification kit for Illumina platforms (Roche, Basel, Switzerland) on a StepOnePlus qPCR machine (Thermo Fisher Scientific, Inc., Dreieich, Germany).

Finally, the pooled libraries were loaded at a concentration of 350 pM with 1% PhiX onto an S2 FlowCell and subjected to paired-end sequencing (2 × 150 bp) using a NovaSeq 6000 next-generation sequencer (Illumina, San Diego, CA, USA). RNA-seq was performed on 13 biological replicates from independent hearts (control n = 7; hypergravity n = 6).

The quality of raw FASTQ sequenced reads was initially evaluated using FastQC v0.11.5 (available at http://www.bioinformatics.babraham.ac.uk/projects/fastqc/) accessed on 5 January 2024. Our sequencing data demonstrated high quality, evidenced by a high Q30 value and a satisfactory mapping rate, indicating a successful sequencing run and reliable base calling. The Supplementary Materials present the base quality (Phred scores) along the length of the reads in each sample. Subsequently, the reads underwent a series of processing steps for quality enhancement and alignment. Initially, Trimmomatic v0.36 was employed to trim reads for quality and remove adapter sequences [90]. The trimmed read pairs were further processed using Fastp to eliminate poly-G tails and address artifacts specific to NovaSeq/NextSeq platforms [91]. Following these quality trimming procedures, the reads were subjected to a second quality assessment using FastQC [92].

After both quality control and trimming, the reads were aligned to the rat reference genome (mRatBN7.2) utilizing HISAT2 with default parameters [93]. The resulting alignments in SAM format were subsequently converted to BAM format and sorted by coordinates using SAM tools v1.3.1 [94]. These sorted alignment files were then processed through HTSeq-count v0.6.1p1 [95] with specific options (-s no -t exon -i gene_id) to generate raw counts for downstream analysis.

Normalization of the raw counts was performed using the TPM (Transcripts per Kilobase Million) method to account for gene length and total read count differences across samples. This step allows for a more accurate comparison of gene expression levels across different genes and samples.

Unless otherwise indicated, all experiments were conducted with a minimum of three replicates, and the presented data are expressed as the mean ± standard deviation (SD).

### 4.6. Western Blot Analysis

The selection of *Trpm7* for protein-level analysis was based on its well-established role in cardiac mechanotransduction and calcium/magnesium homeostasis, as well as prior evidence from our laboratory showing its functional modulation under altered mechanical load conditions. Although *Trpm7* was not identified as significantly differentially expressed in our transcriptomic analysis, it was prioritized due to these established mechanistic links and the availability of validated antibodies for reliable protein detection. TRPM7 protein levels were measured by Western blot analysis. Ventricular cardiomyocytes were lysed with RIPA buffer (Sigma, USA) supplemented with protease and phosphatase inhibitor cocktails (Calbiochem, San Diego, CA, USA). Protein samples were suspended in Laemmli sample buffer supplemented with β-mercaptoethanol, denatured at 95 °C for 5 min, and analyzed. Proteins were resolved using SDS-PAGE at 80 V (stacking gel) and 120 V (resolving gel) and transferred to nitrocellulose membranes. Membranes were incubated with 5% non-fat dry milk in TBS and probed with primary antibodies: anti-TRPM7 (1:1000; A10075, ABclonal, Woburn, MA, USA) and anti-Na^+^/K^+^-ATPase α-subunit (1:100,000; EP1845Y, ab76020, Abcam, Cambridge, UK). Na^+^/K^+^-ATPase α-subunit was chosen as a loading control because, in our RNA-seq dataset, the α1 isoform was among the top stably expressed genes under both control and hypergravity conditions, and its cardiac expression has been reported to remain relatively stable under various mechanical loading paradigms [96]. After washing, the membranes were incubated with HRP-conjugated secondary antibodies (1:10,000; AS014, ABclonal), and the immunoreactive bands were visualized by a ChemiDoc imaging system (Bio-Rad, Hercules, CA, USA). The signal intensity was quantitated by ImageJ version 2.9.0 software (NIH, Bethesda, MD, USA), and TRPM7 expression was normalized to the Na^+^/K^+^ATPase levels [96]. Control cardiomyocytes (n = 11 animals) and hypergravity-exposed cardiomyocytes (n = 10 animals) were studied.

### 4.7. Mechanical Stretch of the Ventricular Myocytes

**Figure 4:**
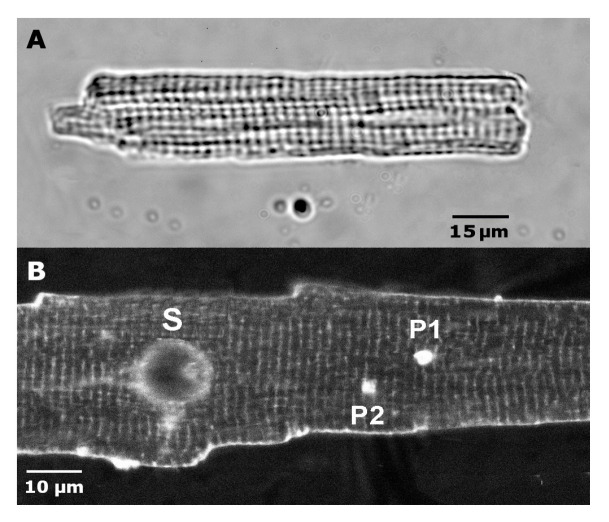
Isolated cardiomyocytes obtained from the ventricle of a rat. (**A**)—Light microscopy of a typical cardiomyocyte of the rat. The sarcomere length is 1.82 µm. (**B**)—Representative images of an isolated cardiomyocyte obtained by membrane fluorescent staining with di-4-ANEPPS (ANEPPS). Positioning of the glass stylus (S) on the surface of the cardiomyocyte, patch-pipette (P1 ~ 40 µm from S), recording in whole-cell mode, and second additional patch-pipette (P2 ~ 30 µm from S), registering (for example) in cell attach mode. Labeled tips of both patch pipettes are visible on the microphotograph as small white spots. The axial stretch of the cardiomyocyte and its release was performed utilizing displacement of the S, the tip of which is visible as a big gray spot. The outer sarcolemma and transversal (T) tubules, stained with ANEPPS, exhibit fluorescence (vertical stripes). The sarcomere length is 1.83 µm.

We have previously described the mechanical stimulation method in detail [7,8]. Here, we report on the specific aspects relevant to this study. Figure 4A displays light microscopy of a typical rat cardiomyocyte. After achieving whole-cell access with a patch pipette (P), a fire-polished glass stylus (S) with a diameter of 14 ± 0.8 µm and a semi-spherical shape [7,8] was attached to the membrane (Figure 4B).

**Figure 5:**
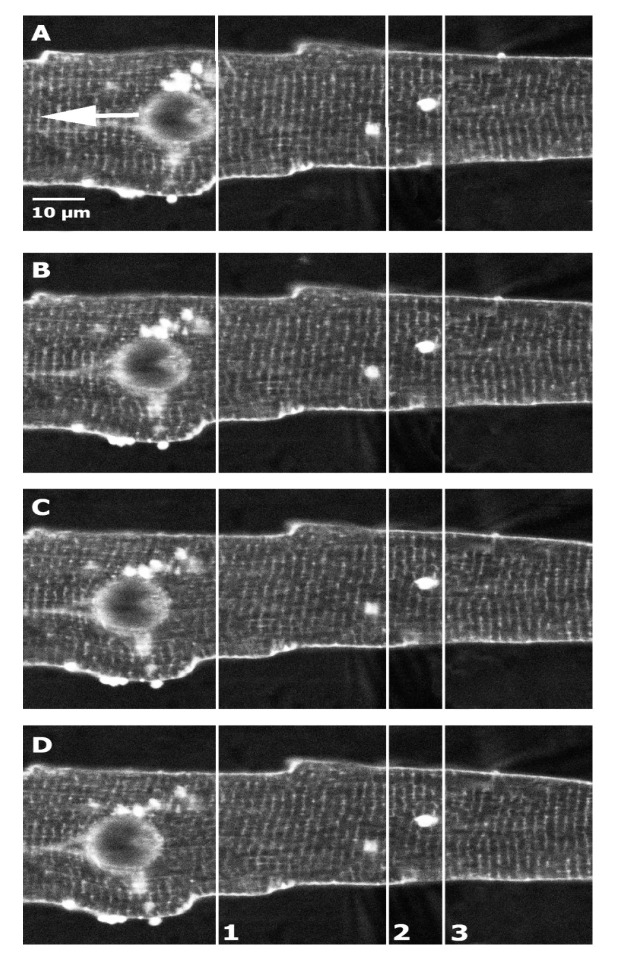
Microphotographs of a cardiomyocyte under control (**A**) and stretching (from resting condition in (**B**–**D**)). The glass stylus (S) is displaced to the left by 4, 8, and 10 µm, while the two patch pipettes, P1 and P2, remain in fixed positions. Sarcomere striation profiles were obtained from regions of interest using ANEPPS images. Fluorescence from ANEPPS-stained membranes highlights the outer sarcolemma and transverse (T) tubules (vertical stripes). Arrows indicate the positions of local maxima in the intensity profiles, visualizing areas near the sarcomeric Z-disks. 1—fixed line for the initial position of the stylus, 2 and 3—fixed lines for the patch pipettes.

The contact area between S and the cell surface was less than 200 μm^2^. The tips of both the S and the P pointed toward each other at a 45° angle relative to the glass bottom [7,8]. The S and P were positioned 40 µm apart before attaching them to the cell. When the S was newly polished and the membrane surface was clean, the attachment was successful in approximately 70% of attempts. The S was then lifted 2 µm to prevent “scratching” the lower cell surface against the coverslip during stretching. A motorized micromanipulator (MP 285, Sutter, Novato, CA, USA, accuracy 0.2 µm) increased the S-P distance stepwise by up to 12 µm, with P remaining fixed [8]. The extent of the local stretch was shown to decay from the cell surface to the interior of the cell, where the optical focus was set [8]. It was assumed that stretching increased the sarcomere length between S and P by bending T-tubular structures [97]. Stretching and releasing stretch could be repeated 3–4 times with the same cell on average (Figure 5).

Sarcomeres are the basic contractile elements of cardiac myocytes, and the actual sarcomere length (SL) is crucial for the whole-cell contractile response. The sarcomere pattern was imaged using an Olympus XM10 camera (Olympus Corporation, Tokyo, Japan) and evaluated with Olympus CellSens software version 3.2 (Olympus Corporation, Tokyo, Japan). The SL of an isolated cardiomyocyte without S and P was calculated based on the distance between sarcomeres lying along a central line in the cell. Before and during cell stretching to fixed lengths, the SL in isolated rat cardiomyocytes was estimated based on the distance between ten sarcomeres along the line connecting S and P.

The variability in individual SLs was analyzed using standard optical images from light microscopy. For ANEPPS measurements, we employed optical images to reveal the regularity of T-tubular invaginations of the cell membrane [98]. Before ANEPPS measurements, cardiomyocytes were labeled with the fluorescent dye di-4-ANEPPS. Specifically, 0.2 μL of ANEPPS (1 mM DMSO stock solution) was added to 1 mL of cell suspension in Tyrode solution at room temperature. After a 10 min dye treatment and sedimentation, the supernatant was removed and replaced with 3 mL of fresh dye-free Tyrode solution. The cells were then placed in a perfusion chamber with a standard perfusion solution stream.

In all measurements, the studied areas of the cell were selected to cover as much of it as possible, with lengths and angles set to follow straight parts of the myofibrils. Curved myofibrils, crossing myofibrils, or myofibrils with irregular staining along their length were excluded from the analysis [98]. The analysis of intracellular variability in SLs was performed using standard protocols for fully relaxed, non-stretched, and stretched cardiomyocytes (Figure 4 and Figure 5).

The method used allows localized stretching of the cell surface (Figure 5). While the membrane along the line between P and S was stretched as intended, approximately 70–80% of the entire surface membrane remained unstretched [7].

### 4.8. Whole-Cell Patch-Clamp

A total of 127 cells (n = 127) from 70 rats (m = 70) were used in the experiments. Whole-cell patch-clamp recordings of the late current (*I*_L_), reflecting *I*_MGC_ or *I*_SAC_, were obtained using an Axopatch 200B amplifier and pClamp 10 software (Molecular Devices, San Jose, CA, USA). The data were filtered at 2 kHz, sampled at 5 kHz, and analyzed using the same software.

The myocytes were superfused in a small recording chamber (RC-26; Warner Instrument Corp., Brunswick, CT, USA) with a volume of 500 µL, which was mounted on an inverted microscope. Freshly isolated, brick-like cardiomyocytes can attach to the glass bottom in two different positions: edgewise, on the narrow side, and broadwise, on the broad side [16,99]. However, the response to stretching was identical in cardiomyocytes occupying both positions. Conversely, the response to compression differs depending on the cell’s position [16,99]. For our experiments, we selected cells that remained on the narrow side (edgewise) and had similar sizes.

Borosilicate glass patch-clamp electrodes had tip resistances ranging from 1.8 to 2.2 MΩ when filled. Cell access was obtained by rupturing the patch after seal formation. To obtain current–voltage relations (*I*/*V* curves), a series of 20 pulses of 140 ms duration at 1 Hz were applied, starting from a holding potential of −45 mV, which caused the inactivation of tetrodotoxin (TTX)-sensitive Na^+^ currents.

Currents in response to trains of short (5 mV) pulses applied at −45 mV were used to assess membrane capacitance and access resistance [7,8,100], without compensation for capacitive and leak currents.

The measurements typically lasted for approximately 20–25 min, during which time access resistance and capacitive current remained stable. To minimize the effects of size differences in the stretched membrane, the glass tools were adjusted to maintain a constant 40 µm S-P distance before applying the stretch. The area of mechanical stretching was small, probably much smaller than 25% of the total membrane area. Since the membrane capacitance measured the entire membrane and not just the stretched portion, and because mechanical stretching was confined to a small area between S and P, we did not normalize the stretch-induced currents (late current: *I*_L_) to the whole membrane capacitance [7].

Local stretch might have induced currents through leakage rather than channels. To test if stretching broke the seal between the patch electrode and the surface membrane, we repeated the stretch experiments with the patch pipette in the cell-attached configuration (n = 6). However, the seal resistance remained constant (1.5 ± 0.3 GΩ before and 1.4 ± 0.4 GΩ during the stretch), and the local stretch did not induce single-channel currents in the cell-attached patch.

Similarly, the access resistance and membrane capacitance remained unaffected, indicating that the stretch-induced inward current was due to the activation of an ionic current rather than leakage around the seal. Hence, the stretch-induced inward current should be attributed to the activation of an ionic current and not to leakage around the seal [7].

In K^+^_in_/K^+^_out_ solutions, net membrane currents at the end of the pulse (“late currents”: *I*_L_) were plotted as functions of the respective clamp step potential. In this context (here and in the works of other authors), “net” means the sum of the individual currents flowing into the cell (negative current) and out of the cell (positive current) in K^+^_in_/K^+^_out_ solutions [7,8]. The intercept of the resulting *I*/*V* curve with the voltage axis defined the zero current potential (*E*_0_), corresponding to the resting membrane potential of a non-clamped cell (between −70 and −80 mV). As strongly demonstrated earlier, net currents in K^+^_in_/K^+^_out_ solutions (and cation non-selective current in Cs^+^_in_/Cs^+^_out_ solutions), which are registered as the late currents (*I*_L_) at the end of the pulse, reflect the cell’s response to stretching [7,8].

To identify the mechanically gated current *I*_SAC_ or ^*C*/*S*^Δ*I_L_*_(−80)_, the differential current at −80 mV was determined. Differential current values were calculated as the difference between control current values (*^C^I_L_*) and current values during cell stretch (*^S^I_L_*) or other actions (results labeled with a Δ) at −80 mV (^*C*/*S*^Δ*I_L_*_(−80)_), designated as Δ*I*_L_ or *I_SAC_* [7,8].

### 4.9. Statistics

Data obtained using the patch-clamp method were processed with the commercial software package Molecular Devices Axon pClamp 10.2. To analyze data within a group of rats, significant differences were determined using a one-way repeated measures analysis of variance (ANOVA for RM) with the Holm–Sidak test as the post hoc test. Statistical significance was set at *p* < 0.05. The normality of sample distributions was verified using the Shapiro–Wilk test. The data were presented as the mean ± standard error of the mean (SEM), with n representing the number of experiments and m representing the number of rat hearts. To analyze intergroup differences, we used two-way analysis of variance (ANOVA).

In the study, RNA-seq was used to examine the TPMs of channel genes, with m = 3 in the control group and m = 3 after hypergravity exposure. For SL, the control group had n = 16 and m = 8, while after hypergravity exposure, there were n = 10 and m = 4. When examining changes in SL under stretch, the control group had n = 35 and m = 16, and after hypergravity exposure, there were n = 20 and m = 12. In the study of changes in *I_SAC_* due to stretch magnitude, the control group had n = 75 and m = 34, while after hypergravity exposure, there were n = 52 and m = 36.

## 5. Conclusions and Limitations

### 5.1. Conclusions

Several channel-encoding genes were identified as mechanosensitive, and their expression was apparently remodeled under hypergravity conditions. *Trpm7* was identified as a candidate in which changes were observed consistently at the mRNA and protein levels, which confirmed its role in hypergravity-altered mechanotransduction. Although additional channel genes (*Trpc1*, *Traak*/*Kcnk4*, *Kir6.2*/*Kcnj11*, *Piezo2*, and *Trpv2*) exhibited differential expression, their protein levels were not assessed in this study, and thus these findings should be regarded as preliminary, requiring further functional and protein-level validation.

Consistent with hypertrophic development and enhanced mechanical coupling between the cytoskeleton and sarcolemma, these molecular changes were complemented by structural modifications, including significant sarcomere elongation and a 44% increase in membrane capacitance. Patch-clamp analysis demonstrated that stretch-activated currents were markedly hypersensitive, with enhanced responses to various mechanical stimuli and currents elicited at minimal displacements. The combined data indicate that hypergravity activates protective mechanisms to preserve electrical stability while reducing the threshold for mechanical activation.

These coordinated transcriptional, structural, and electrophysiological changes underscore the adaptability of cardiac mechanotransduction under sustained mechanical stress. These alterations may enhance mechanoelectric feedback and contractile efficiency; nevertheless, they also pose a risk of inducing arrhythmogenesis if stabilizing systems are overwhelmed. Our findings therefore highlight that hypergravity induces remodeling not only of pore-forming channel genes but also of critical auxiliary subunits (with the suppression of *Kcnmb1*), pointing to an important layer of regulatory adaptation.

This study provides new insights into the heart’s adaptations to altered gravitational environments, with implications for understanding hypertrophy, arrhythmia risk, and cardiovascular deconditioning in aerospace settings or under prolonged mechanical loading on Earth.

### 5.2. Limitations

Several limitations must be considered when interpreting the present findings. First, the transcriptome analysis was performed on a limited sample size, potentially restricting the statistical power to detect more subtle changes in gene expression. Some channels exhibited considerable but statistically insignificant fold changes, suggesting that biological significance may exceed transcript-level statistical relevance. Second, we did not thoroughly validate all transcriptome findings at the protein or functional level. We confirmed TRPM7 expression using Western blot analysis and functionally assessed stretch-activated currents. However, other candidates such as TRAAK and PIEZO2 require further protein-level and electrophysiological assessments to establish a direct correlation with the observed remodeling. Third, the complex interactions between subunits, exemplified by Kir6.1 and Kir6.2, were mostly inferred from transcript data, without direct assessment of channel architecture or ATP sensitivity in native tissue. Fourth, our research design focused on relatively short-term (14-day) hypergravity exposure; the persistence, enhancement, or reversal of these adaptations over extended durations remains unknown. Finally, although the rat model provides valuable insights into physiological mechanisms, caution is warranted when extrapolating these findings to human cardiac physiology due to interspecies variations in ion channel expression and mechanotransductive pathways that may influence outcomes.

These limitations highlight the need for future studies employing multi-omics approaches, targeted interventions, and extended time-course analyses to thoroughly elucidate the full spectrum of cardiac remodeling under hypergravity conditions.

## Figures and Tables

**Table 1:** Percentage of hypergravity-induced changes in mechanically gated channels.

Mechanically Gated Channels (MGCs)	References	Control	Hypergravity	% of Change	*p*
*Trpm7*	[22,23,24,25]	0.2362 ± 0.0214	0.3336 ± 0.0249	41.23%	0.0073
*Trpc1*	[26]	0.2049 ± 0.0240	0.3447 ± 0.0317	68.23%	0.0026
*Trpm4*	[27,28,29]	0.2699 ± 0.0083	0.2380 ± 0.0132	−11.80%	0.0562
*Trpv2*	[30]	0.0679 ± 0.0108	0.0257 ± 0.0067	−62.19%	0.0044
*Trpc3*	[31,32,33,34]	0.0151 ± 0.0051	0.0069 ± 0.0007	−54.06%	0.1392
*Trpv4*	[35,36,37]	0.0111 ± 0.0028	0.0086 ± 0.0029	−22.77%	0.5100
*Trpv1*	[38,39]	0.0053 ± 0.0012	0.0063 ± 0.0006	18.24%	0.4609
*Trpm3*	[40]	0.0001 ± 0.0001	0.0001 ± 0.0001	N/A	N/A
*Trpc5*	[41]	0.0002 ± 0.0001	0.0003 ± 0.0001	55.56%	0.1663
*Trpa1*	[42,43,44,45]	0.0009 ± 0.0009	0.0003 ± 0.0002	−62.96%	0.5306
*Trpc6*	[27,46,47]	0	0	N/A	N/A
*Pkd1* (TRPP1)	[48]	0.6161 ± 0.1106	0.6562 ± 0.0315	6.50%	0.5884
*Pkd2* (TRPP2)	[49,50]	0.2889 ± 0.0486	0.2887 ± 0.0447	−0.07%	0.9796
*Piezo1*	[51,52,53,54,55]	0.1630 ± 0.0484	0.1064 ± 0.0161	−34.73%	0.2954
*Piezo2*	[55,56]	0.0028 ± 0.0005	0.0012 ± 0.0001	−57.58%	0.0079
*Tmem63a*		0.0960 ± 0.0253	0.1234 ± 0.0330	28.50%	0.4695
*Tmem63b*		2.5330 ± 0.3292	2.9867 ± 0.4486	17.91%	0.5046
*Kcnk2* (*Trek1*/K2P2.1)	[57,58,59,60]	0.0868 ± 0.0150	0.1061 ± 0.0074	22.14%	0.2372
*Kcnk4* (*Traak*/K2P4.1)	[59,61]	0.0035 ± 0.0003	0.0118 ± 0.0022	239.48%	0.0092
*Kcnk10* (*Trek2*/K2P10.1)	[59]	0	0.0002 ± 0.0001	N/A	N/A
*Kcnma1* (BK_Ca_ subunit)		0.0001 ± 0.0001	0.0001 ± 0.0001	N/A	N/A
*Kcnmb1* (BK_Ca_ subunit)	[62,63]	0.0120 ± 0.0039	0.0063 ± 0.0034	−47.84%	0.0203
*Kcnmb2* (BK_Ca_ subunit)	[62,63]	0.0107 ± 0.0013	0.0124 ± 0.0025	16.20%	0.5206
*Kcnmb4* (BK_Ca_ subunit)	[62,63]	0.0111 ± 0.0035	0.0053 ± 0.0012	−52.29%	0.1291
*Scn5a* (Na_V_1.5)	[64,65]	3.3240 ± 0.5076	3.9881 ± 0.6835	19.98%	0.7391
*Scn8a* (Na_V_1.6)	[66]	0	0	N/A	N/A
*Cacna1c* (Ca_V_1.2, L-type)	[12]	0.1850 ± 0.0242	0.2865 ± 0.0530	54.85%	0.0868
*Cacna1d* (Ca_V_1.3, L-type)	[13]	0.0016 ± 0.0002	0.0016 ± 0.0001	−1.92%	0.8910
*Cacna1b* (Ca_V_2.2, N-type)	[67]	0	0.0001 ± 0.0001	N/A	N/A
*Kcnq1* (Kv7.1/KCNQ1)	[47,68,69]	0.2237 ± 0.0296	0.2666 ± 0.0346	19.15%	0.3380
*Kcnj11* (Kir6.2)	[70]	35.2505 ± 4.0662	48.8510 ± 4.2813	38.58%	0.0317
*Kcnj8* (Kir6.1)	[71]	2.7115 ± 0.9426	0.6555 ± 0.1284	−75.83%	0.0085

Percent change represents the relative increase or decrease in TPM values compared to the control condition. N/A indicates that no transcriptional expression data are available in the literature. *p* < 0.05 was considered statistically significant.

## Data Availability

The data that support the findings of this study are available in Gene Expression Omnibus (GEO) at https://www.ncbi.nlm.nih.gov/geo/query/acc.cgi?acc=GSE306309 accessed on 5 January 2024. All further data gathered in this study (including manual patch-clamp measurements and their analysis procedures) are available from the corresponding author upon request.

## Abbreviations

The following abbreviations are used in this manuscript:

ANEPPS	4-(2-(6-(dioctylamino)-2-naphthalenyl)ethenyl)-1-(3-sulfopropyl)-pyridinium
ATP	Adenosine Triphosphate
BK_Ca_	Big Potassium (Large Conductance Ca^2+^-Activated K^+^) Channel
Ca^2+^	Calcium ion
Ca_V_1.2/Ca_V_1.3/Ca_V_2.1/Ca_V_2.2	Voltage-gated calcium channel subtypes
Cs^+^_in_/Cs^+^_out_	Intracellular/extracellular cesium ion configuration
g	Gravitational acceleration
*I*_L_	Late Membrane Current
*I*_SAC_	Stretch-Activated Current
K^+^_in_/K^+^_out_	Intracellular/extracellular potassium ion configuration
K_ATP_	ATP-sensitive potassium channel
KB	Kraftbrühe solution (storage medium)
*K*_ir_	Inward Rectifier Potassium Channel
*K*_V_	Voltage-Gated Potassium Channel
L	Number of measured sarcomeres
MGCs	Mechanically Gated Channels
MGCK	K^+^-selective Mechanically Gated Channels
MSC	Mechanosensitive Channel
Na_V_	Voltage-gated Sodium Channel
P	Patch pipette
P_Ca_/P_Na_	Relative permeability of Ca^2+^ vs. Na^+^
PKD1/PKD2 (TRPP1/TRPP2)	Polycystin-1 and Polycystin-2 (mechanosensitive TRP channels)
RNA-seq	RNA Sequencing
S	Stylus (glass stylus for mechanical stimulation)
SAKCA	Stretch-Activated, Ca^2+^-activated K^+^ channel (alternative for BK_Ca_)
SCN2A	Gene encoding Na_V_1.2
SHR	Spontaneously Hypertensive Rat
SL	Sarcomere Length
TPM	Transcripts per Kilobase Million
TRP	Transient Receptor Potential channel
TRPC/TRPV/TRPM/TRPA/TRPP/TMEM63	Subtypes of TRP ion channels
TREK/TRAAK	Two-pore-domain K^+^ channels (K_2_P family)
TWIK-1	Tandem of P domains in a Weak Inward rectifying K^+^ channel
VGCs	Voltage-Gated Channels
VGCMS	Voltage-Gated Channels with Mechanosensitivity
V_0_	Zero current potential (resting membrane potential)
WKY	Wistar-Kyoto Rat
